# Crystal structures of (*E*)-1-{3-[(5-fluoro-2-hy­droxy­benzyl­idene)amino]­phen­yl}ethanone and of a fourth polymorph of (*E*)-1-{3-[(2-hy­droxy-3-meth­oxy­benzyl­idene)amino]­phen­yl}ethanone

**DOI:** 10.1107/S2056989017015985

**Published:** 2017-11-07

**Authors:** Marisiddaiah Girisha, Hemmige S. Yathirajan, Ravindranath S. Rathore, Christopher Glidewell

**Affiliations:** a Department of Studies in Chemistry, University of Mysore, Manasagangotri, Mysuru 570 006, India; b Centre for Biological Sciences (Bioinformatics), School of Earth, Biological and Environmental Sciences, Central University of South Bihar, Patna 800 014, India; c School of Chemistry, University of St Andrews, St Andrews, Fife KY16 9ST, UK

**Keywords:** crystal structure, mol­ecular conformation, hydrogen bonding, polymorphism

## Abstract

In the mol­ecules of both (*E*)-1-{3-[(5-fluoro-2-hy­droxy­benzyl­idene)amino]­phen­yl}ethanone, C_15_H_12_FNO_2_, (I), and (*E*)-1-{3-[(2-hy­droxy-3-meth­oxy­benzyl­idene)amino]­phen­yl}ethanone, C_16_H_15_NO_3_, (II), which crystallizes with *Z*′ = 2 in space group *Pca*2_1_, there are intra­molecular O—H⋯N hydrogen bonds, and the non-H atoms in each mol­ecule are essentially coplanar. In the crystal of (I), mol­ecules are linked by a single C—H⋯O hydrogen bond to form a *C*(8) chain, whereas in the crystal of (II), mol­ecules are linked by three C—H⋯O hydrogen bonds to form sheets within which orthogonal *C*_2_^2^(16) and *C*_2_^2^(17) chains can be identified. Comparisons are made with some related structures.

## Abstract 2

The mol­ecules of the title compounds are effectively planar, apart from the methyl H atoms. In the crystals, C—H⋯O hydrogen bonds link the mol­ecules into chains in one compound and into sheets in the other.

## Chemical context   

**Figure 1:**
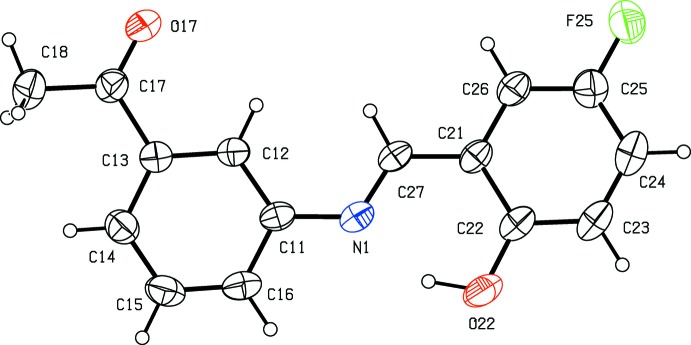
The mol­ecular structure of compound (I), showing the atom-labelling scheme. Displacement ellipsoids are drawn at the 30% probability level.

**Figure 2:**
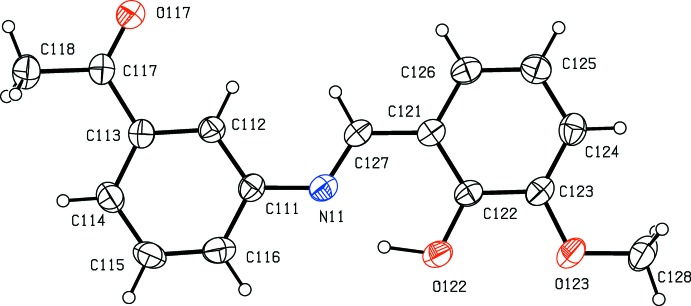
The structure of mol­ecule 1 in compound (II), showing the atom-labelling scheme. Displacement ellipsoids are drawn at the 30% probability level.

Schiff bases of general type *RR*′C=N*R*′′ can exhibit very wide structural diversity and have found a wide range of applications (Jia & Li, 2015 ▸), ranging from anti-bacterial, anti-fungal and anti-tumour activity (Rani *et al.*, 2015 ▸), *via* catalysis (Kumar *et al.*, 2009 ▸), to use as organic photovoltaic materials (Jeevadason *et al.*, 2014 ▸). The extensive patent literature on their medicinal applications has recently been reviewed (Hameed *et al.*, 2017 ▸). With this great diversity of use in mind, we report herein on the mol­ecular and supra­molecular structures of two closely related Schiff bases,(*E*)-1-{3-[(5-fluoro-2-hy­droxy­benzyl­idene)amino]­phen­yl}ethanone (I) and (*E*)-1-{3-[(2-hy­droxy-3-meth­oxy­benzyl­idene)amino]­phen­yl}ethanone (II). Compounds (I) and (II) were prepared by straightforward condensation reactions between 3-acetyl­aniline (3-amino­aceto­phenone) and the appropriately substituted salicyl­aldehydes. Their mol­ecular constitutions differ only in the identity and location of a single substituent, 5-fluoro in (I) *versus* 3-meth­oxy in (II), but their crystallization behaviour is different. Compound (I) crystallizes in the monoclinic space group *P*2_1_/*n* with *Z*′ = 1 (Fig. 1 ▸), while compound (II) crystallizes in the ortho­rhom­bic space group *Pca*2_1_ with *Z*′ = 2 (Figs. 2 ▸ and 3 ▸), and it will be convenient to refer to the mol­ecules of (II) which contain the atoms N11 and N21 as mol­ecules of types 1 and 2, respectively. Compound (II), in fact, represents the fourth polymorphic form of this compound to be identified. Three other forms, one in *Pna*2_1_ with *Z*′ = 2, and two others in *P*2_1_2_1_2_1_, each with *Z*′ = 1, have recently been reported (Zbačnik *et al.*, 2015 ▸).
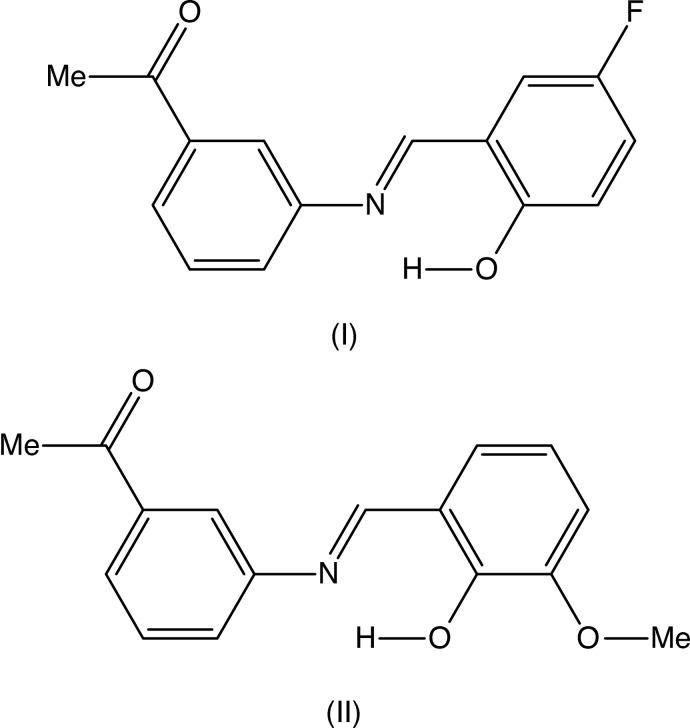


## Structural commentary   

In each of compounds (I) (Fig. 1 ▸) and (II) (Figs. 2 ▸ and 3 ▸), the non-H atoms are almost coplanar. Thus in (I), the r.m.s. deviation of the non-H atoms from their mean plane is only 0.085 Å, with a maximum individual deviation from the plane of 0.196 (2) Å for the acetyl atom C18. Similarly, in compound (II), the r.m.s. deviations of the non-H atoms from the mean planes of the two mol­ecules are 0.086 and 0.071 Å for mol­ecules 1 and 2, respectively, with corresponding maximum deviations of 0.225 (5) and 0.211 (5) Å for atoms C118 and C218, respectively. In all of the mol­ecules there is an intra­molecular O—H⋯N hydrogen bond (Tables 1 ▸ and 2 ▸); although this probably influences the orientation of the hy­droxy­lated ring relative to the central spacer unit, it will not have any influence on the orientation of the acetyl­phenyl ring relative to the rest of the mol­ecule. In the two mol­ecules of (II), the deviation of the meth­oxy C atoms C128 and C228 from the planes of their adjacent aryl rings are 0.107 (9) and 0.049 (11) Å, respectively. Consistent with this, the pair of exocyclic C—C—O angles at each of the atoms C123 and C223 differ by *ca* 10°, as is generally observed in planar alk­oxy­arene derivatives (Seip & Seip, 1973 ▸; Ferguson *et al.*, 1996 ▸). The dihedral angle between the mean planes of the two mol­ecules in (II) is 80.74 (3)°.

## Supra­molecular features   

**Figure 4:**
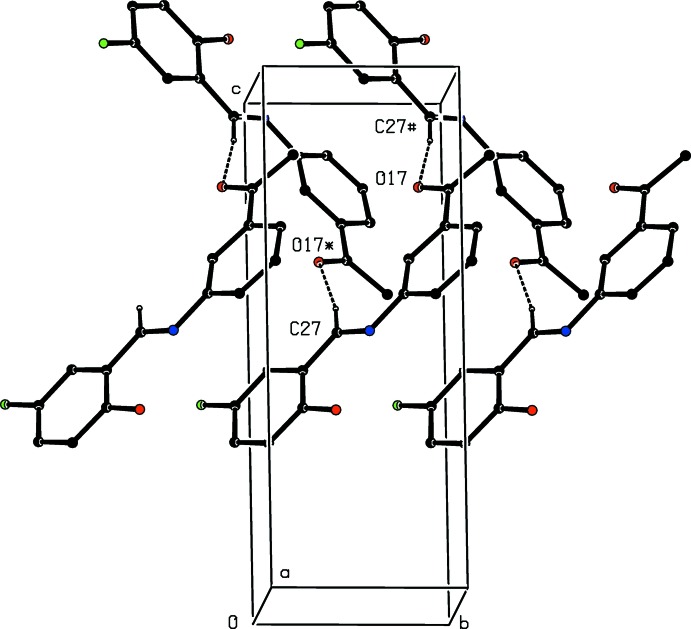
Part of the crystal structure of compound (I), showing the formation of a hydrogen-bonded *C*(8) chain running parallel to the [010] direction. For the sake of clarity, the H atoms not involved in the motif shown have been omitted. Hydrogen bonds are drawn as dashed lines and the atoms marked with an asterisk (*) or a hash (#) are at the symmetry positions (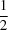 − *x*, −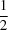 + *y*, 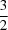 − *z*) and (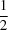 − *x*, 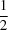 + *y*, 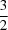 − *z*), respectively.

The supra­molecular assembly in compound (I) is very simple, as shown in Fig. 4 ▸. In addition to the intra­molecular hydrogen bond noted above, there is a single C—H⋯O hydrogen bond (Table 1 ▸), which links mol­ecules related by a 2_1_ screw axis into *C*(8)chains running parallel to the [010] direction. Two chains of this type, related to one another by inversion, pass through each unit cell, but there are no direction-specific inter­actions between adjacent chains.

**Figure 5:**
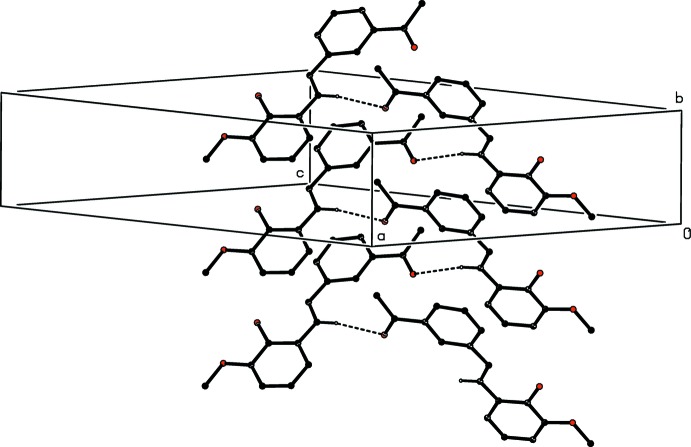
Part of the crystal structure of compound (II), showing the formation of a hydrogen-bonded 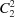
(16) chain running parallel to the [010] direction. For the sake of clarity, the H atoms not involved in the motif shown have been omitted, and the hydrogen bonds are drawn as dashed lines.

**Figure 6:**
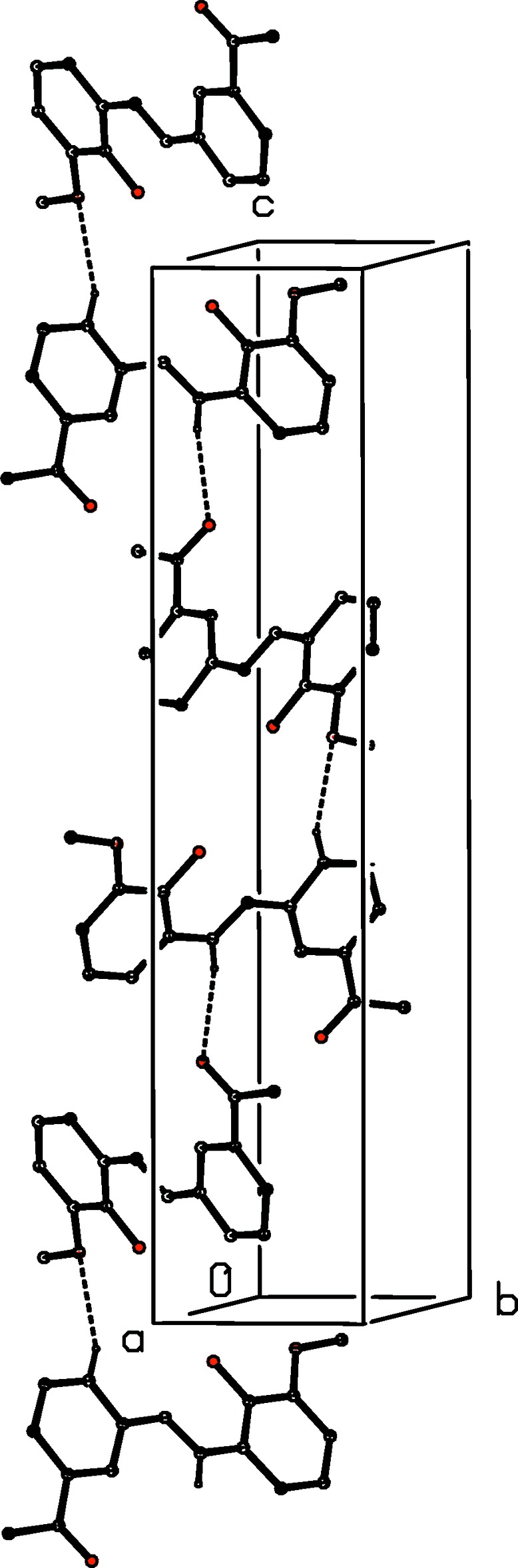
Part of the crystal structure of compound (II), showing the formation of a hydrogen-bonded 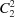
(17) chain running parallel to the [001] direction. For the sake of clarity, the H atoms not involved in the motif shown have been omitted, and the hydrogen bonds are drawn as dashed lines.

There are three C—H⋯O hydrogen bonds in the structure of compound (II) (Table 2 ▸): one of these links the two mol­ecules within the selected asymmetric unit and the two others link these bimolecular aggregates into complex sheets, whose formation is readily analysed in terms of two one-dimensional sub-structures (Ferguson *et al.*, 1998*a* ▸,*b* ▸; Gregson *et al.*, 2000 ▸). The hydrogen bond having atom C227 as the donor links bimolecular aggregates related by translation to form a 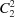
(16) chain running parallel to the [010] direction (Fig. 5 ▸), and that having atom C116 as the donor links aggregates related by a 2_1_ screw axis into 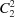
(17) chains running parallel to the [001] direction (Fig. 6 ▸). The combination of the orthogonal chains along [010] and [001] generates a sheet lying parallel to (100). Two sheets of this type, related to one another by the glide planes, pass through each unit cell but there are no direction-specific inter­actions between adjacent sheets.

## Database survey   

The structures of Schiff bases derived from hydroxyaryl aldehydes have recently been the subject of a general survey, in which a number of structural errors, often involving misplaced H atoms, were pointed out (Blagus *et al.*, 2010 ▸). Closely related to the present structures are those of (*E*)-1-{3-[(2-hy­droxy-3-meth­oxy­benzyl­idene)amino]­phen­yl}ethanone (III) (De *et al.*, 2009 ▸), and of the previously recorded polymorphs of (II) (Zbačnik *et al.*, 2015 ▸).

Compound (III) is isomorphous with compound (I): as in (I), the structure of (III) contains an intra­molecular O—H⋯N hydrogen bond and the non-H atoms are effectively coplanar. The structure of (III) also contains an inter­molecular C—H⋯O hydrogen bond, although this is nowhere mentioned in the original report (De *et al.*, 2009 ▸); this inter­action forms *C*(8) chains along [010], exactly the same as those in the structure of (I), so that (I) and (III) are, in fact, isostructural despite their different patterns of substitution.

Three other polymorphic forms of compound (II) have recently been reported and are described as forms I, II and II,I respectively (Zbačnik *et al.*, 2015 ▸). Form I is ortho­rhom­bic in space group *Pna*2_1_ with *Z*′ = 2, and forms II and III both crystallize in space group *P*2_1_2_1_2_1_ with *Z*′ = 1, so that the *Pca*2_1_ form reported here can be regarded as form IV. All three forms, I–III, can be crystallized from ethanol solutions under different conditions and a crucial factor in determining which polymorph is obtained appears to be the filtration process used prior to crystallization. By contrast, the form described here was crystallized from a solution in di­chloro­methane. In all of the mol­ecules in forms I–III, there is an intra­molecular O—H⋯N hydrogen bond and, in every case, the non-H atoms are effectively co-planar as found here for (I) and (II). The supra­molecular assembly differs in all three polymorphs I–III: form II contains no inter­molecular hydrogen bonds; in form III two C—H⋯O hydrogen bonds generate a *C*(8)*C*(10)[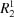
(6)] chain of rings; and in form I, three C—H⋯O hydrogen bonds generate sheets in which the component sub-structures both involve mol­ecules related by an *n*-glide plane, in contrast to the sheets found for form IV reported here.

## Synthesis and crystallization   

For the synthesis of compounds (I) and (II), 3-acetyl aniline (0.740 mmol) and a catalytic qu­antity of acetic acid were added to solution of the appropriate aldehyde, 5-fluoro­salicyl­aldehyde for (I) or 3-meth­oxy­salicyl­aldehyde for (II) (0.740 mmol) in ethanol (20 cm^3^), and these mixtures were then heated under reflux for 5 h. The mixtures were then cooled to ambient temperature and the solvent was removed under reduced pressure. The solid residues were then washed with cold ethanol and dried under reduced pressure. Crystals suitable for single crystal X-ray diffraction were grown by slow evaporation, at ambient temperature and in the presence of air, of solutions in di­methyl­sulfoxide for (I) and in di­chloro­methane for (II): m.p. for (I) 362–364 K and m.p. for (II) 352–354 K.

## Refinement   

Crystal data, data collection and structure refinement details are summarized in Table 3 ▸. For compound (II), one bad outlier reflection (8,1,3) was omitted from the data set before the final refinements. All H atoms were located in difference-Fourier maps. The C-bound H atoms were subsequently treated as riding atoms in geometrically idealized positions: C—H 0.93–0.96 Å with *U*_iso_(H) = 1.5*U*_eq_(C-meth­yl) and 1.2*U*_eq_(C) for other C-bound H atoms. The methyl groups were permitted to rotate but not to tilt. For the H atoms bonded to O atoms, the atomic coordinates were refined with *U*_iso_(H) = 1.5*U*_eq_(O), giving the O—H distances shown in Tables 1 ▸ and 2 ▸. The correct orientation of the structure of (II) relative to the polar axis direction was established by means of the Flack *x* parameter (Flack, 1983 ▸), *x* = −0.04 (16) calculated (Parsons *et al.*, 2013 ▸) using 1493 quotients of the type [(*I* ^+^)−(*I* ^−^)]/[(*I* ^+^)+(*I* ^−^)], and by means of the Hooft y parameter (Hooft *et al.*, 2010 ▸), *y* = −0.03 (16). In the final analysis of variance for (I) there was a large value, 1.859, of *K* = [mean(*F*_o_^2^)/mean(*F*_c_^2^)] for the group of 4258 very weak reflections having *F*_c_/*F*_c_(max) in the range 0.000 < *F*_c_/*F*_c_(max) < 0.008; the corresponding value for (II) was 2.1539 for 565 reflections having *F*_c_/*F*_c_(max) in the range 0.000 < *F*_c_/*F*_c_(max) < 0.009.

## Supplementary Material

Crystal structure: contains datablock(s) global, I, II. DOI: 10.1107/S2056989017015985/su5402sup1.cif

Structure factors: contains datablock(s) I. DOI: 10.1107/S2056989017015985/su5402Isup2.hkl

Structure factors: contains datablock(s) II. DOI: 10.1107/S2056989017015985/su5402IIsup3.hkl

Supporting information file. DOI: 10.1107/S2056989017015985/su5402Isup4.cml

Supporting information file. DOI: 10.1107/S2056989017015985/su5402IIsup5.cml

CCDC references: 1583686, 1583685

Additional supporting information:  crystallographic information; 3D view; checkCIF report

## Figures and Tables

**Figure 3:**
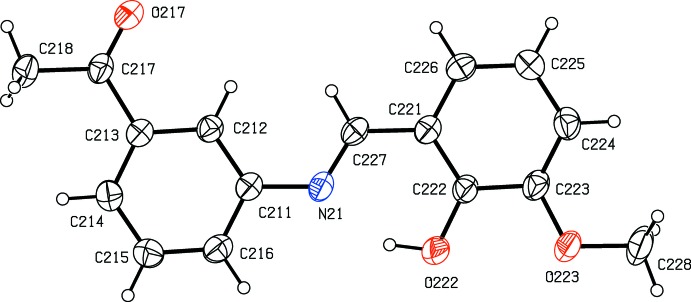
The structure of mol­ecule 2 in compound (II), showing the atom-labelling scheme. Displacement ellipsoids are drawn at the 30% probability level.

**Table 1:** Hydrogen-bond geometry (Å, °) for (I)

*D*—H⋯*A*	*D*—H	H⋯*A*	*D*⋯*A*	*D*—H⋯*A*
O22—H22⋯N1	0.98 (3)	1.72 (3)	2.607 (2)	148 (3)
C27—H27⋯O17^i^	0.93	2.58	3.475 (3)	163

Symmetry code: (i) 
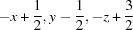
.

**Table 2:** Hydrogen-bond geometry (Å, °) for (II)

*D*—H⋯*A*	*D*—H	H⋯*A*	*D*⋯*A*	*D*—H⋯*A*
O122—H122⋯N11	1.06 (6)	1.68 (6)	2.604 (4)	142 (5)
O222—H222⋯N21	0.92 (6)	1.79 (6)	2.603 (5)	147 (5)
C116—H116⋯O223^i^	0.93	2.50	3.347 (6)	152
C127—H127⋯O217	0.93	2.59	3.496 (5)	164
C227—H227⋯O117^ii^	0.93	2.58	3.487 (5)	164

Symmetry codes: (i) 
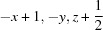
; (ii) 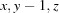.

**Table 3:** Experimental details

	(I)	(II)
Crystal data		
Chemical formula	C_15_H_12_FNO_2_	C_16_H_15_NO_3_
*M*_r_	257.26	269.29
Crystal system, space group	Monoclinic, *P*2_1_/*n*	Orthorhombic, *P* *c* *a*2_1_
Temperature (K)	294	294
*a*, *b*, *c* (Å)	14.9527 (5), 5.5152 (2), 16.6918 (5)	19.1904 (4), 5.33856 (12), 26.5678 (6)
α, β, γ (°)	90, 114.739 (2), 90	90, 90, 90
*V* (Å^3^)	1250.19 (7)	2721.85 (10)
*Z*	4	8
Radiation type	Cu *K*α	Cu *K*α
μ (mm^−1^)	0.84	0.75
Crystal size (mm)	0.15 × 0.15 × 0.10	0.10 × 0.10 × 0.05

Data collection		
Diffractometer	Bruker APEX3	Bruker APEX3
Absorption correction	Multi-scan (*SADABS*; Bruker, 2016 ▸)	Multi-scan (*SADABS*; Bruker, 2016 ▸)
*T*_min_, *T*_max_	0.848, 0.919	0.907, 0.963
No. of measured, independent and observed [*I* > 2σ(*I*)] reflections	15703, 2452, 1764	51776, 5393, 3796
*R*_int_	0.041	0.117
(sin θ/λ)_max_ (Å^−1^)	0.619	0.619

Refinement		
*R*[*F* ^2^ > 2σ(*F* ^2^)], *wR*(*F* ^2^), *S*	0.046, 0.122, 1.04	0.048, 0.113, 1.02
No. of reflections	2452	5393
No. of parameters	176	371
No. of restraints	0	1
H-atom treatment	H atoms treated by a mixture of independent and constrained refinement	H atoms treated by a mixture of independent and constrained refinement
Δρ_max_, Δρ_min_ (e Å^−3^)	0.15, −0.12	0.11, −0.13
Absolute structure	–	Flack *x* determined using 1493 quotients [(*I* ^+^)−(*I* ^−^)]/[(*I* ^+^)+(*I* ^−^)] (Parsons *et al.*, 2013 ▸)
Absolute structure parameter	–	−0.04 (16)

Computer programs: *APEX3* and *SAINT* (Bruker, 2016 ▸), *SHELXT2014* (Sheldrick, 2015*a* ▸), *SHELXL2014* (Sheldrick, 2015*b* ▸) and *PLATON* (Spek, 2009 ▸).

## supplementary crystallographic information

### (E)-1-{3-[(5-Fluoro-2-hydroxybenzylidene)amino]phenyl}ethanone (I) Crystal data

**Table:**

C_15_H_12_FNO_2_	*F*(000) = 536
*M**_r_* = 257.26	*D*_x_ = 1.367 Mg m^−^^3^
Monoclinic, *P*2_1_/*n*	Cu *K*α radiation, λ = 1.54178 Å
*a* = 14.9527 (5) Å	Cell parameters from 2452 reflections
*b* = 5.5152 (2) Å	θ = 3.3–72.5°
*c* = 16.6918 (5) Å	µ = 0.84 mm^−^^1^
β = 114.739 (2)°	*T* = 294 K
*V* = 1250.19 (7) Å^3^	Block, yellow
*Z* = 4	0.15 × 0.15 × 0.10 mm

### (E)-1-{3-[(5-Fluoro-2-hydroxybenzylidene)amino]phenyl}ethanone (I) Data collection

**Table:**

Bruker APEX3 diffractometer	2452 independent reflections
Radiation source: microfocus sealed tube	1764 reflections with *I* > 2σ(*I*)
Multilayer mirror monochromator	*R*_int_ = 0.041
φ and ω scans	θ_max_ = 72.5°, θ_min_ = 3.3°
Absorption correction: multi-scan (SADABS; Bruker, 2016)	*h* = −18→18
*T*_min_ = 0.848, *T*_max_ = 0.919	*k* = −6→6
15703 measured reflections	*l* = −20→20

### (E)-1-{3-[(5-Fluoro-2-hydroxybenzylidene)amino]phenyl}ethanone (I) Refinement

**Table:**

Refinement on *F*^2^	Primary atom site location: structure-invariant direct methods
Least-squares matrix: full	Secondary atom site location: difference Fourier map
*R*[*F*^2^ > 2σ(*F*^2^)] = 0.046	Hydrogen site location: mixed
*wR*(*F*^2^) = 0.122	H atoms treated by a mixture of independent and constrained refinement
*S* = 1.03	*w* = 1/[σ^2^(*F*_o_^2^) + (0.0427*P*)^2^ + 0.3677*P*] where *P* = (*F*_o_^2^ + 2*F*_c_^2^)/3
2452 reflections	(Δ/σ)_max_ < 0.001
176 parameters	Δρ_max_ = 0.15 e Å^−^^3^
0 restraints	Δρ_min_ = −0.12 e Å^−^^3^

### (E)-1-{3-[(5-Fluoro-2-hydroxybenzylidene)amino]phenyl}ethanone (I) Special details

**Table:**

Geometry. All esds (except the esd in the dihedral angle between two l.s. planes) are estimated using the full covariance matrix. The cell esds are taken into account individually in the estimation of esds in distances, angles and torsion angles; correlations between esds in cell parameters are only used when they are defined by crystal symmetry. An approximate (isotropic) treatment of cell esds is used for estimating esds involving l.s. planes.

### (E)-1-{3-[(5-Fluoro-2-hydroxybenzylidene)amino]phenyl}ethanone (I) Fractional atomic coordinates and isotropic or equivalent isotropic displacement parameters (Å^2^)

**Table:**

	*x*	*y*	*z*	*U*_iso_*/*U*_eq_	
N1	0.35562 (11)	0.5843 (3)	0.53906 (9)	0.0566 (4)	
C11	0.39602 (12)	0.7592 (3)	0.60710 (11)	0.0529 (4)	
C12	0.36799 (12)	0.7897 (3)	0.67598 (11)	0.0539 (4)	
H12	0.3219	0.6851	0.6812	0.065*	
C13	0.40781 (12)	0.9740 (3)	0.73703 (11)	0.0551 (4)	
C14	0.47738 (14)	1.1282 (4)	0.72986 (13)	0.0672 (5)	
H14	0.5041	1.2531	0.7704	0.081*	
C15	0.50686 (15)	1.0962 (4)	0.66268 (14)	0.0750 (6)	
H15	0.5540	1.1987	0.6583	0.090*	
C16	0.46702 (14)	0.9138 (4)	0.60227 (12)	0.0655 (5)	
H16	0.4878	0.8932	0.5574	0.079*	
C17	0.37489 (14)	0.9972 (4)	0.80988 (12)	0.0635 (5)	
O17	0.31869 (12)	0.8495 (3)	0.81717 (10)	0.0859 (5)	
C18	0.41085 (18)	1.2043 (5)	0.87234 (15)	0.0900 (7)	
H18A	0.3840	1.1931	0.9153	0.135*	
H18B	0.3903	1.3539	0.8404	0.135*	
H18C	0.4814	1.1995	0.9016	0.135*	
C27	0.29372 (13)	0.4249 (3)	0.53959 (10)	0.0554 (4)	
H27	0.2759	0.4216	0.5867	0.066*	
C21	0.25071 (13)	0.2505 (3)	0.46968 (10)	0.0541 (4)	
C22	0.27440 (15)	0.2459 (4)	0.39638 (12)	0.0632 (5)	
O22	0.33757 (13)	0.4080 (3)	0.38812 (10)	0.0858 (5)	
H22	0.361 (2)	0.512 (5)	0.4408 (19)	0.129*	
C23	0.23177 (18)	0.0728 (4)	0.33148 (13)	0.0774 (6)	
H23	0.2473	0.0695	0.2830	0.093*	
C24	0.16706 (17)	−0.0935 (4)	0.33765 (13)	0.0776 (6)	
H24	0.1386	−0.2094	0.2938	0.093*	
C25	0.14467 (15)	−0.0872 (4)	0.40930 (13)	0.0701 (5)	
F25	0.08044 (11)	−0.2522 (3)	0.41531 (9)	0.1050 (5)	
C26	0.18467 (14)	0.0810 (4)	0.47482 (11)	0.0631 (5)	
H26	0.1678	0.0819	0.5225	0.076*	

### (E)-1-{3-[(5-Fluoro-2-hydroxybenzylidene)amino]phenyl}ethanone (I) Atomic displacement parameters (Å^2^)

**Table:**

	*U*^11^	*U*^22^	*U*^33^	*U*^12^	*U*^13^	*U*^23^
N1	0.0650 (9)	0.0630 (9)	0.0489 (8)	0.0097 (7)	0.0308 (7)	0.0060 (7)
C11	0.0543 (9)	0.0585 (10)	0.0503 (9)	0.0096 (8)	0.0263 (7)	0.0124 (8)
C12	0.0550 (9)	0.0571 (10)	0.0556 (9)	−0.0021 (8)	0.0290 (8)	0.0028 (8)
C13	0.0542 (9)	0.0569 (10)	0.0556 (10)	0.0015 (8)	0.0243 (8)	0.0026 (8)
C14	0.0680 (12)	0.0636 (11)	0.0681 (11)	−0.0095 (9)	0.0266 (9)	0.0000 (9)
C15	0.0710 (13)	0.0798 (14)	0.0795 (13)	−0.0147 (11)	0.0366 (11)	0.0115 (12)
C16	0.0664 (11)	0.0795 (13)	0.0614 (11)	0.0038 (10)	0.0372 (9)	0.0150 (10)
C17	0.0646 (11)	0.0673 (12)	0.0634 (11)	0.0009 (10)	0.0314 (9)	−0.0068 (9)
O17	0.1074 (11)	0.0916 (11)	0.0868 (10)	−0.0230 (9)	0.0682 (9)	−0.0232 (8)
C18	0.0975 (16)	0.0946 (17)	0.0870 (15)	−0.0151 (14)	0.0476 (13)	−0.0330 (13)
C27	0.0641 (10)	0.0641 (11)	0.0434 (8)	0.0101 (9)	0.0279 (8)	0.0076 (8)
C21	0.0636 (10)	0.0573 (10)	0.0416 (8)	0.0157 (8)	0.0222 (7)	0.0076 (7)
C22	0.0782 (12)	0.0663 (11)	0.0528 (10)	0.0190 (10)	0.0349 (9)	0.0058 (9)
O22	0.1131 (12)	0.0961 (11)	0.0753 (9)	−0.0030 (9)	0.0660 (9)	−0.0058 (8)
C23	0.1021 (16)	0.0819 (14)	0.0559 (11)	0.0201 (13)	0.0406 (11)	−0.0040 (11)
C24	0.0948 (15)	0.0731 (13)	0.0556 (11)	0.0175 (12)	0.0222 (10)	−0.0091 (10)
C25	0.0775 (13)	0.0646 (12)	0.0601 (11)	0.0036 (10)	0.0209 (10)	0.0051 (10)
F25	0.1246 (11)	0.1000 (10)	0.0836 (9)	−0.0353 (9)	0.0367 (8)	−0.0144 (8)
C26	0.0735 (12)	0.0698 (12)	0.0454 (9)	0.0064 (10)	0.0245 (8)	0.0051 (9)

### (E)-1-{3-[(5-Fluoro-2-hydroxybenzylidene)amino]phenyl}ethanone (I) Geometric parameters (Å, º)

**Table:**

N1—C27	1.279 (2)	C18—H18B	0.9600
N1—C11	1.418 (2)	C18—H18C	0.9600
C11—C12	1.389 (2)	C27—C21	1.440 (2)
C11—C16	1.390 (2)	C27—H27	0.9300
C12—C13	1.385 (2)	C21—C26	1.388 (3)
C12—H12	0.9300	C21—C22	1.409 (2)
C13—C14	1.387 (2)	C22—O22	1.349 (2)
C13—C17	1.496 (2)	C22—C23	1.383 (3)
C14—C15	1.377 (3)	O22—H22	0.98 (3)
C14—H14	0.9300	C23—C24	1.368 (3)
C15—C16	1.371 (3)	C23—H23	0.9300
C15—H15	0.9300	C24—C25	1.371 (3)
C16—H16	0.9300	C24—H24	0.9300
C17—O17	1.213 (2)	C25—F25	1.357 (2)
C17—C18	1.487 (3)	C25—C26	1.366 (3)
C18—H18A	0.9600	C26—H26	0.9300
			
C27—N1—C11	121.93 (14)	C17—C18—H18C	109.5
C12—C11—C16	118.43 (17)	H18A—C18—H18C	109.5
C12—C11—N1	124.77 (15)	H18B—C18—H18C	109.5
C16—C11—N1	116.78 (15)	N1—C27—C21	122.19 (15)
C13—C12—C11	120.82 (16)	N1—C27—H27	118.9
C13—C12—H12	119.6	C21—C27—H27	118.9
C11—C12—H12	119.6	C26—C21—C22	119.03 (17)
C12—C13—C14	119.56 (16)	C26—C21—C27	119.20 (15)
C12—C13—C17	118.34 (16)	C22—C21—C27	121.76 (17)
C14—C13—C17	122.09 (17)	O22—C22—C23	119.35 (17)
C15—C14—C13	119.92 (19)	O22—C22—C21	121.21 (17)
C15—C14—H14	120.0	C23—C22—C21	119.4 (2)
C13—C14—H14	120.0	C22—O22—H22	107.5 (17)
C16—C15—C14	120.28 (18)	C24—C23—C22	120.82 (18)
C16—C15—H15	119.9	C24—C23—H23	119.6
C14—C15—H15	119.9	C22—C23—H23	119.6
C15—C16—C11	120.97 (17)	C23—C24—C25	119.2 (2)
C15—C16—H16	119.5	C23—C24—H24	120.4
C11—C16—H16	119.5	C25—C24—H24	120.4
O17—C17—C18	120.53 (17)	F25—C25—C26	118.85 (18)
O17—C17—C13	120.10 (17)	F25—C25—C24	119.1 (2)
C18—C17—C13	119.37 (18)	C26—C25—C24	122.1 (2)
C17—C18—H18A	109.5	C25—C26—C21	119.48 (17)
C17—C18—H18B	109.5	C25—C26—H26	120.3
H18A—C18—H18B	109.5	C21—C26—H26	120.3
			
C27—N1—C11—C12	−5.5 (3)	C11—N1—C27—C21	178.53 (15)
C27—N1—C11—C16	176.20 (16)	N1—C27—C21—C26	179.41 (16)
C16—C11—C12—C13	1.8 (2)	N1—C27—C21—C22	−0.1 (3)
N1—C11—C12—C13	−176.54 (16)	C26—C21—C22—O22	179.49 (17)
C11—C12—C13—C14	−0.7 (3)	C27—C21—C22—O22	−1.0 (3)
C11—C12—C13—C17	−179.86 (16)	C26—C21—C22—C23	−0.2 (3)
C12—C13—C14—C15	−0.5 (3)	C27—C21—C22—C23	179.31 (17)
C17—C13—C14—C15	178.60 (18)	O22—C22—C23—C24	−179.71 (18)
C13—C14—C15—C16	0.6 (3)	C21—C22—C23—C24	0.0 (3)
C14—C15—C16—C11	0.4 (3)	C22—C23—C24—C25	0.0 (3)
C12—C11—C16—C15	−1.6 (3)	C23—C24—C25—F25	179.98 (19)
N1—C11—C16—C15	176.83 (17)	C23—C24—C25—C26	0.3 (3)
C12—C13—C17—O17	3.8 (3)	F25—C25—C26—C21	179.80 (16)
C14—C13—C17—O17	−175.26 (19)	C24—C25—C26—C21	−0.5 (3)
C12—C13—C17—C18	−175.50 (18)	C22—C21—C26—C25	0.5 (3)
C14—C13—C17—C18	5.4 (3)	C27—C21—C26—C25	−179.09 (16)

### (E)-1-{3-[(5-Fluoro-2-hydroxybenzylidene)amino]phenyl}ethanone (I) Hydrogen-bond geometry (Å, º)

**Table:**

*D*—H···*A*	*D*—H	H···*A*	*D*···*A*	*D*—H···*A*
O22—H22···N1	0.98 (3)	1.72 (3)	2.607 (2)	148 (3)
C27—H27···O17^i^	0.93	2.58	3.475 (3)	163

Symmetry code: (i) −*x*+1/2, *y*−1/2, −*z*+3/2.

### (E)-1-{3-[(2-Hydroxy-3-methoxybenzylidene)amino]phenyl}ethanone (II) Crystal data

**Table:**

C_16_H_15_NO_3_	*D*_x_ = 1.314 Mg m^−^^3^
*M**_r_* = 269.29	Cu *K*α radiation, λ = 1.54184 Å
Orthorhombic, *P**c**a*2_1_	Cell parameters from 5394 reflections
*a* = 19.1904 (4) Å	θ = 3.3–72.6°
*b* = 5.33856 (12) Å	µ = 0.75 mm^−^^1^
*c* = 26.5678 (6) Å	*T* = 294 K
*V* = 2721.85 (10) Å^3^	Block, yellow
*Z* = 8	0.10 × 0.10 × 0.05 mm
*F*(000) = 1136	

### (E)-1-{3-[(2-Hydroxy-3-methoxybenzylidene)amino]phenyl}ethanone (II) Data collection

**Table:**

Bruker APEX3 diffractometer	5393 independent reflections
Radiation source: microfocus sealed tube	3796 reflections with *I* > 2σ(*I*)
Multilayer mirror monochromator	*R*_int_ = 0.117
φ and ω scans	θ_max_ = 72.6°, θ_min_ = 3.3°
Absorption correction: multi-scan (SADABS; Bruker, 2016)	*h* = −23→23
*T*_min_ = 0.907, *T*_max_ = 0.963	*k* = −6→6
51776 measured reflections	*l* = −32→32

### (E)-1-{3-[(2-Hydroxy-3-methoxybenzylidene)amino]phenyl}ethanone (II) Refinement

**Table:**

Refinement on *F*^2^	Secondary atom site location: difference Fourier map
Least-squares matrix: full	Hydrogen site location: mixed
*R*[*F*^2^ > 2σ(*F*^2^)] = 0.048	H atoms treated by a mixture of independent and constrained refinement
*wR*(*F*^2^) = 0.113	*w* = 1/[σ^2^(*F*_o_^2^) + (0.0463*P*)^2^ + 0.4368*P*] where *P* = (*F*_o_^2^ + 2*F*_c_^2^)/3
*S* = 1.02	(Δ/σ)_max_ < 0.001
5393 reflections	Δρ_max_ = 0.11 e Å^−^^3^
371 parameters	Δρ_min_ = −0.13 e Å^−^^3^
1 restraint	Absolute structure: Flack *x* determined using 1493 quotients [(*I*^+^)-(*I*^-^)]/[(*I*^+^)+(*I*^-^)] (Parsons *et al.*, 2013)
Primary atom site location: structure-invariant direct methods	Absolute structure parameter: −0.04 (16)

### (E)-1-{3-[(2-Hydroxy-3-methoxybenzylidene)amino]phenyl}ethanone (II) Special details

**Table:**

Geometry. All esds (except the esd in the dihedral angle between two l.s. planes) are estimated using the full covariance matrix. The cell esds are taken into account individually in the estimation of esds in distances, angles and torsion angles; correlations between esds in cell parameters are only used when they are defined by crystal symmetry. An approximate (isotropic) treatment of cell esds is used for estimating esds involving l.s. planes.

### (E)-1-{3-[(2-Hydroxy-3-methoxybenzylidene)amino]phenyl}ethanone (II) Fractional atomic coordinates and isotropic or equivalent isotropic displacement parameters (Å^2^)

**Table:**

	*x*	*y*	*z*	*U*_iso_*/*U*_eq_	
N11	0.73276 (17)	0.3158 (7)	0.39187 (12)	0.0502 (8)	
C111	0.6779 (2)	0.4896 (8)	0.38707 (15)	0.0466 (10)	
C112	0.6379 (2)	0.5213 (8)	0.34388 (15)	0.0475 (10)	
H112	0.6453	0.4169	0.3164	0.057*	
C113	0.5874 (2)	0.7053 (8)	0.34117 (15)	0.0485 (10)	
C114	0.5762 (3)	0.8623 (9)	0.38243 (18)	0.0610 (11)	
H114	0.5431	0.9893	0.3807	0.073*	
C115	0.6145 (3)	0.8276 (10)	0.42579 (18)	0.0673 (13)	
H115	0.6066	0.9297	0.4536	0.081*	
C116	0.6641 (2)	0.6437 (9)	0.42814 (17)	0.0613 (12)	
H116	0.6891	0.6212	0.4578	0.074*	
C117	0.5463 (2)	0.7282 (9)	0.29372 (17)	0.0535 (11)	
O117	0.55324 (18)	0.5744 (7)	0.26015 (13)	0.0739 (10)	
C118	0.4965 (3)	0.9408 (11)	0.28736 (19)	0.0750 (15)	
H11A	0.4741	0.9280	0.2552	0.112*	
H11B	0.5214	1.0965	0.2893	0.112*	
H11C	0.4620	0.9346	0.3135	0.112*	
C127	0.7464 (2)	0.1542 (8)	0.35777 (15)	0.0495 (10)	
H127	0.7185	0.1499	0.3292	0.059*	
C121	0.8030 (2)	−0.0210 (8)	0.36174 (14)	0.0453 (10)	
C122	0.8458 (2)	−0.0276 (8)	0.40460 (14)	0.0459 (9)	
C123	0.8976 (2)	−0.2119 (9)	0.40885 (15)	0.0520 (11)	
C124	0.9068 (2)	−0.3823 (9)	0.37035 (16)	0.0577 (11)	
H124	0.9407	−0.5059	0.3733	0.069*	
C125	0.8660 (2)	−0.3715 (9)	0.32720 (17)	0.0584 (11)	
H125	0.8737	−0.4843	0.3011	0.070*	
C126	0.8145 (2)	−0.1951 (8)	0.32308 (16)	0.0555 (11)	
H126	0.7870	−0.1906	0.2943	0.067*	
O122	0.83809 (16)	0.1383 (6)	0.44236 (10)	0.0596 (8)	
H122	0.798 (3)	0.267 (10)	0.433 (2)	0.089*	
O123	0.93514 (16)	−0.2049 (7)	0.45239 (12)	0.0740 (10)	
C128	0.9848 (3)	−0.3972 (10)	0.4596 (2)	0.0769 (15)	
H12A	0.9620	−0.5571	0.4583	0.115*	
H12B	1.0068	−0.3765	0.4917	0.115*	
H12C	1.0193	−0.3886	0.4335	0.115*	
N21	0.49089 (17)	−0.1872 (7)	0.10711 (13)	0.0520 (9)	
C211	0.5456 (2)	−0.0084 (9)	0.11255 (15)	0.0477 (10)	
C212	0.58545 (19)	0.0190 (9)	0.15570 (15)	0.0486 (10)	
H212	0.5781	−0.0874	0.1829	0.058*	
C213	0.6363 (2)	0.2045 (8)	0.15866 (15)	0.0482 (10)	
C214	0.6471 (2)	0.3607 (9)	0.11801 (17)	0.0579 (11)	
H214	0.6803	0.4875	0.1199	0.069*	
C215	0.6087 (2)	0.3286 (9)	0.07452 (17)	0.0634 (12)	
H215	0.6170	0.4309	0.0468	0.076*	
C216	0.5582 (2)	0.1458 (9)	0.07194 (17)	0.0589 (12)	
H216	0.5324	0.1262	0.0426	0.071*	
C217	0.6772 (2)	0.2248 (9)	0.20644 (17)	0.0540 (11)	
O217	0.67061 (18)	0.0695 (7)	0.23932 (13)	0.0769 (10)	
C218	0.7275 (3)	0.4370 (10)	0.21282 (19)	0.0759 (15)	
H21A	0.7023	0.5924	0.2138	0.114*	
H21B	0.7595	0.4390	0.1851	0.114*	
H21C	0.7528	0.4158	0.2437	0.114*	
C227	0.4769 (2)	−0.3452 (9)	0.14214 (16)	0.0494 (11)	
H227	0.5037	−0.3431	0.1713	0.059*	
C221	0.4212 (2)	−0.5259 (9)	0.13805 (15)	0.0483 (10)	
C222	0.3792 (2)	−0.5367 (9)	0.09519 (15)	0.0544 (11)	
C223	0.3269 (2)	−0.7193 (10)	0.09223 (16)	0.0629 (13)	
C224	0.3180 (3)	−0.8857 (9)	0.13109 (18)	0.0626 (13)	
H224	0.2842	−1.0098	0.1286	0.075*	
C225	0.3588 (2)	−0.8708 (9)	0.17392 (18)	0.0600 (12)	
H225	0.3517	−0.9833	0.2002	0.072*	
C226	0.4092 (2)	−0.6926 (8)	0.17789 (17)	0.0553 (11)	
H226	0.4358	−0.6813	0.2071	0.066*	
O222	0.38787 (19)	−0.3768 (8)	0.05600 (12)	0.0797 (12)	
H222	0.425 (3)	−0.274 (12)	0.063 (2)	0.119*	
O223	0.28893 (19)	−0.7128 (8)	0.04865 (12)	0.0957 (14)	
C228	0.2377 (3)	−0.9052 (14)	0.0424 (2)	0.109 (2)	
H22A	0.2603	−1.0655	0.0410	0.163*	
H22B	0.2125	−0.8774	0.0117	0.163*	
H22C	0.2059	−0.9018	0.0703	0.163*	

### (E)-1-{3-[(2-Hydroxy-3-methoxybenzylidene)amino]phenyl}ethanone (II) Atomic displacement parameters (Å^2^)

**Table:**

	*U*^11^	*U*^22^	*U*^33^	*U*^12^	*U*^13^	*U*^23^
N11	0.0449 (19)	0.061 (2)	0.0451 (19)	−0.0023 (18)	−0.0030 (15)	0.0026 (18)
C111	0.038 (2)	0.052 (3)	0.050 (2)	−0.003 (2)	0.0001 (16)	0.0036 (19)
C112	0.051 (3)	0.048 (2)	0.044 (2)	0.001 (2)	−0.0012 (17)	0.0011 (18)
C113	0.046 (2)	0.046 (2)	0.054 (2)	−0.0002 (19)	0.0016 (18)	0.004 (2)
C114	0.062 (3)	0.054 (3)	0.067 (3)	0.008 (2)	0.003 (2)	−0.008 (2)
C115	0.071 (3)	0.072 (3)	0.059 (3)	0.002 (3)	0.002 (2)	−0.024 (2)
C116	0.060 (3)	0.075 (3)	0.049 (2)	−0.007 (3)	−0.005 (2)	−0.007 (2)
C117	0.050 (2)	0.051 (3)	0.060 (3)	0.006 (2)	0.001 (2)	0.005 (2)
O117	0.090 (2)	0.069 (2)	0.063 (2)	0.0239 (19)	−0.0219 (17)	−0.0097 (19)
C118	0.077 (3)	0.075 (4)	0.074 (3)	0.024 (3)	−0.004 (3)	0.004 (3)
C127	0.045 (2)	0.056 (3)	0.047 (2)	−0.004 (2)	−0.0084 (18)	0.005 (2)
C121	0.043 (2)	0.048 (2)	0.045 (2)	−0.008 (2)	−0.0013 (17)	0.006 (2)
C122	0.045 (2)	0.053 (2)	0.040 (2)	−0.006 (2)	0.0003 (16)	0.0062 (18)
C123	0.043 (2)	0.065 (3)	0.047 (2)	−0.003 (2)	−0.0026 (18)	0.011 (2)
C124	0.049 (2)	0.062 (3)	0.062 (3)	0.004 (2)	0.004 (2)	0.010 (2)
C125	0.059 (3)	0.057 (3)	0.059 (3)	−0.004 (2)	0.001 (2)	−0.002 (2)
C126	0.052 (2)	0.062 (3)	0.053 (3)	−0.008 (2)	−0.007 (2)	−0.001 (2)
O122	0.0591 (18)	0.075 (2)	0.0451 (16)	0.0062 (16)	−0.0082 (14)	−0.0004 (15)
O123	0.069 (2)	0.097 (3)	0.0564 (19)	0.024 (2)	−0.0184 (17)	0.0036 (18)
C128	0.065 (3)	0.089 (4)	0.077 (4)	0.016 (3)	−0.018 (3)	0.014 (3)
N21	0.0439 (19)	0.062 (2)	0.050 (2)	−0.0086 (18)	−0.0011 (15)	−0.0127 (18)
C211	0.042 (2)	0.053 (3)	0.048 (2)	0.003 (2)	−0.0002 (17)	−0.0074 (19)
C212	0.041 (2)	0.058 (3)	0.046 (2)	−0.001 (2)	0.0011 (17)	−0.0033 (19)
C213	0.044 (2)	0.053 (2)	0.048 (2)	0.003 (2)	−0.0008 (17)	−0.0063 (19)
C214	0.053 (3)	0.055 (3)	0.065 (3)	−0.006 (2)	0.001 (2)	0.001 (2)
C215	0.065 (3)	0.063 (3)	0.063 (3)	−0.002 (3)	−0.004 (2)	0.010 (2)
C216	0.054 (3)	0.069 (3)	0.054 (3)	0.000 (2)	−0.009 (2)	−0.002 (2)
C217	0.052 (2)	0.055 (3)	0.054 (3)	−0.007 (2)	−0.003 (2)	−0.012 (2)
O217	0.088 (3)	0.078 (2)	0.064 (2)	−0.023 (2)	−0.0262 (18)	0.005 (2)
C218	0.082 (4)	0.078 (3)	0.067 (3)	−0.031 (3)	−0.010 (3)	−0.011 (3)
C227	0.038 (2)	0.059 (3)	0.051 (3)	0.006 (2)	−0.0052 (18)	−0.008 (2)
C221	0.039 (2)	0.058 (3)	0.047 (2)	0.002 (2)	0.0019 (17)	−0.010 (2)
C222	0.047 (2)	0.073 (3)	0.043 (2)	−0.015 (2)	0.0048 (17)	−0.007 (2)
C223	0.055 (3)	0.089 (4)	0.044 (2)	−0.021 (3)	0.005 (2)	−0.015 (2)
C224	0.060 (3)	0.067 (3)	0.061 (3)	−0.016 (2)	0.012 (2)	−0.010 (2)
C225	0.061 (3)	0.058 (3)	0.061 (3)	−0.001 (2)	0.005 (2)	0.004 (2)
C226	0.052 (2)	0.059 (3)	0.054 (2)	0.007 (2)	−0.007 (2)	−0.001 (2)
O222	0.079 (2)	0.117 (3)	0.0437 (17)	−0.045 (2)	−0.0040 (16)	0.004 (2)
O223	0.093 (3)	0.143 (4)	0.0508 (18)	−0.067 (3)	−0.0126 (18)	0.000 (2)
C228	0.097 (4)	0.151 (6)	0.078 (4)	−0.070 (4)	−0.010 (3)	−0.022 (4)

### (E)-1-{3-[(2-Hydroxy-3-methoxybenzylidene)amino]phenyl}ethanone (II) Geometric parameters (Å, º)

**Table:**

N11—C127	1.278 (5)	N21—C227	1.285 (5)
N11—C111	1.409 (5)	N21—C211	1.427 (5)
C111—C112	1.391 (5)	C211—C216	1.378 (6)
C111—C116	1.392 (6)	C211—C212	1.386 (5)
C112—C113	1.382 (6)	C212—C213	1.393 (6)
C112—H112	0.9300	C212—H212	0.9300
C113—C114	1.397 (6)	C213—C214	1.380 (6)
C113—C117	1.492 (6)	C213—C217	1.497 (6)
C114—C115	1.379 (7)	C214—C215	1.381 (6)
C114—H114	0.9300	C214—H214	0.9300
C115—C116	1.369 (6)	C215—C216	1.378 (6)
C115—H115	0.9300	C215—H215	0.9300
C116—H116	0.9300	C216—H216	0.9300
C117—O117	1.219 (5)	C217—O217	1.211 (5)
C117—C118	1.494 (7)	C217—C218	1.497 (6)
C118—H11A	0.9600	C218—H21A	0.9600
C118—H11B	0.9600	C218—H21B	0.9600
C118—H11C	0.9600	C218—H21C	0.9600
C127—C121	1.437 (5)	C227—C221	1.443 (6)
C127—H127	0.9300	C227—H227	0.9300
C121—C126	1.403 (6)	C221—C222	1.397 (6)
C121—C122	1.405 (5)	C221—C226	1.402 (6)
C122—O122	1.346 (5)	C222—O222	1.357 (6)
C122—C123	1.403 (6)	C222—C223	1.401 (6)
C123—O123	1.363 (5)	C223—O223	1.369 (5)
C123—C124	1.380 (6)	C223—C224	1.373 (7)
C124—C125	1.389 (6)	C224—C225	1.384 (7)
C124—H124	0.9300	C224—H224	0.9300
C125—C126	1.369 (6)	C225—C226	1.361 (6)
C125—H125	0.9300	C225—H225	0.9300
C126—H126	0.9300	C226—H226	0.9300
O122—H122	1.06 (5)	O222—H222	0.93 (6)
O123—C128	1.414 (5)	O223—C228	1.432 (6)
C128—H12A	0.9600	C228—H22A	0.9600
C128—H12B	0.9600	C228—H22B	0.9600
C128—H12C	0.9600	C228—H22C	0.9600
			
C127—N11—C111	122.2 (4)	C227—N21—C211	121.3 (4)
C112—C111—C116	118.0 (4)	C216—C211—C212	119.2 (4)
C112—C111—N11	124.5 (4)	C216—C211—N21	116.7 (4)
C116—C111—N11	117.4 (4)	C212—C211—N21	124.1 (4)
C113—C112—C111	121.1 (4)	C211—C212—C213	120.5 (4)
C113—C112—H112	119.4	C211—C212—H212	119.7
C111—C112—H112	119.4	C213—C212—H212	119.7
C112—C113—C114	119.6 (4)	C214—C213—C212	119.4 (4)
C112—C113—C117	118.2 (4)	C214—C213—C217	122.7 (4)
C114—C113—C117	122.2 (4)	C212—C213—C217	117.9 (4)
C115—C114—C113	119.5 (4)	C213—C214—C215	120.0 (4)
C115—C114—H114	120.3	C213—C214—H214	120.0
C113—C114—H114	120.3	C215—C214—H214	120.0
C116—C115—C114	120.4 (4)	C216—C215—C214	120.3 (4)
C116—C115—H115	119.8	C216—C215—H215	119.8
C114—C115—H115	119.8	C214—C215—H215	119.8
C115—C116—C111	121.4 (4)	C215—C216—C211	120.5 (4)
C115—C116—H116	119.3	C215—C216—H216	119.8
C111—C116—H116	119.3	C211—C216—H216	119.8
O117—C117—C113	120.4 (4)	O217—C217—C213	120.5 (4)
O117—C117—C118	119.9 (4)	O217—C217—C218	120.3 (4)
C113—C117—C118	119.7 (4)	C213—C217—C218	119.3 (4)
C117—C118—H11A	109.5	C217—C218—H21A	109.5
C117—C118—H11B	109.5	C217—C218—H21B	109.5
H11A—C118—H11B	109.5	H21A—C218—H21B	109.5
C117—C118—H11C	109.5	C217—C218—H21C	109.5
H11A—C118—H11C	109.5	H21A—C218—H21C	109.5
H11B—C118—H11C	109.5	H21B—C218—H21C	109.5
N11—C127—C121	122.8 (4)	N21—C227—C221	122.6 (4)
N11—C127—H127	118.6	N21—C227—H227	118.7
C121—C127—H127	118.6	C221—C227—H227	118.7
C126—C121—C122	119.0 (4)	C222—C221—C226	119.7 (4)
C126—C121—C127	119.8 (4)	C222—C221—C227	121.1 (4)
C122—C121—C127	121.2 (4)	C226—C221—C227	119.3 (4)
O122—C122—C123	118.6 (3)	O222—C222—C221	122.0 (4)
O122—C122—C121	121.5 (4)	O222—C222—C223	118.8 (4)
C123—C122—C121	119.8 (4)	C221—C222—C223	119.2 (4)
O123—C123—C124	125.4 (4)	O223—C223—C224	125.9 (4)
O123—C123—C122	115.1 (4)	O223—C223—C222	114.3 (4)
C124—C123—C122	119.5 (4)	C224—C223—C222	119.8 (4)
C123—C124—C125	120.8 (4)	C223—C224—C225	120.7 (4)
C123—C124—H124	119.6	C223—C224—H224	119.6
C125—C124—H124	119.6	C225—C224—H224	119.6
C126—C125—C124	120.1 (4)	C226—C225—C224	120.4 (4)
C126—C125—H125	120.0	C226—C225—H225	119.8
C124—C125—H125	120.0	C224—C225—H225	119.8
C125—C126—C121	120.8 (4)	C225—C226—C221	120.1 (4)
C125—C126—H126	119.6	C225—C226—H226	119.9
C121—C126—H126	119.6	C221—C226—H226	119.9
C122—O122—H122	109 (3)	C222—O222—H222	108 (4)
C123—O123—C128	116.8 (4)	C223—O223—C228	116.5 (4)
O123—C128—H12A	109.5	O223—C228—H22A	109.5
O123—C128—H12B	109.5	O223—C228—H22B	109.5
H12A—C128—H12B	109.5	H22A—C228—H22B	109.5
O123—C128—H12C	109.5	O223—C228—H22C	109.5
H12A—C128—H12C	109.5	H22A—C228—H22C	109.5
H12B—C128—H12C	109.5	H22B—C228—H22C	109.5
			
C127—N11—C111—C112	−6.6 (6)	C227—N21—C211—C216	177.4 (4)
C127—N11—C111—C116	174.9 (4)	C227—N21—C211—C212	−3.2 (6)
C116—C111—C112—C113	2.0 (6)	C216—C211—C212—C213	1.8 (6)
N11—C111—C112—C113	−176.5 (4)	N21—C211—C212—C213	−177.6 (4)
C111—C112—C113—C114	−0.1 (6)	C211—C212—C213—C214	−0.4 (6)
C111—C112—C113—C117	−179.9 (4)	C211—C212—C213—C217	179.7 (4)
C112—C113—C114—C115	−1.6 (7)	C212—C213—C214—C215	−1.5 (7)
C117—C113—C114—C115	178.2 (4)	C217—C213—C214—C215	178.5 (4)
C113—C114—C115—C116	1.2 (8)	C213—C214—C215—C216	1.8 (7)
C114—C115—C116—C111	0.9 (7)	C214—C215—C216—C211	−0.4 (7)
C112—C111—C116—C115	−2.4 (6)	C212—C211—C216—C215	−1.4 (7)
N11—C111—C116—C115	176.2 (4)	N21—C211—C216—C215	178.1 (4)
C112—C113—C117—O117	6.3 (6)	C214—C213—C217—O217	−173.1 (4)
C114—C113—C117—O117	−173.5 (4)	C212—C213—C217—O217	6.9 (6)
C112—C113—C117—C118	−173.7 (4)	C214—C213—C217—C218	5.9 (7)
C114—C113—C117—C118	6.5 (7)	C212—C213—C217—C218	−174.2 (4)
C111—N11—C127—C121	178.8 (4)	C211—N21—C227—C221	179.1 (4)
N11—C127—C121—C126	179.4 (4)	N21—C227—C221—C222	−0.2 (6)
N11—C127—C121—C122	1.6 (6)	N21—C227—C221—C226	179.9 (4)
C126—C121—C122—O122	178.5 (4)	C226—C221—C222—O222	178.8 (4)
C127—C121—C122—O122	−3.7 (6)	C227—C221—C222—O222	−1.0 (7)
C126—C121—C122—C123	−2.1 (6)	C226—C221—C222—C223	−1.8 (6)
C127—C121—C122—C123	175.7 (4)	C227—C221—C222—C223	178.4 (4)
O122—C122—C123—O123	0.8 (6)	O222—C222—C223—O223	−0.6 (7)
C121—C122—C123—O123	−178.6 (4)	C221—C222—C223—O223	180.0 (4)
O122—C122—C123—C124	−179.4 (4)	O222—C222—C223—C224	178.9 (5)
C121—C122—C123—C124	1.2 (6)	C221—C222—C223—C224	−0.5 (7)
O123—C123—C124—C125	−179.4 (4)	O223—C223—C224—C225	−178.7 (5)
C122—C123—C124—C125	0.9 (7)	C222—C223—C224—C225	1.9 (7)
C123—C124—C125—C126	−2.0 (7)	C223—C224—C225—C226	−1.0 (7)
C124—C125—C126—C121	1.0 (7)	C224—C225—C226—C221	−1.4 (7)
C122—C121—C126—C125	1.0 (6)	C222—C221—C226—C225	2.8 (7)
C127—C121—C126—C125	−176.8 (4)	C227—C221—C226—C225	−177.4 (4)
C124—C123—O123—C128	−3.7 (7)	C224—C223—O223—C228	−3.2 (8)
C122—C123—O123—C128	176.1 (4)	C222—C223—O223—C228	176.2 (5)

### (E)-1-{3-[(2-Hydroxy-3-methoxybenzylidene)amino]phenyl}ethanone (II) Hydrogen-bond geometry (Å, º)

**Table:**

*D*—H···*A*	*D*—H	H···*A*	*D*···*A*	*D*—H···*A*
O122—H122···N11	1.06 (6)	1.68 (6)	2.604 (4)	142 (5)
O222—H222···N21	0.92 (6)	1.79 (6)	2.603 (5)	147 (5)
C116—H116···O223^i^	0.93	2.50	3.347 (6)	152
C127—H127···O217	0.93	2.59	3.496 (5)	164
C227—H227···O117^ii^	0.93	2.58	3.487 (5)	164

Symmetry codes: (i) −*x*+1, −*y*, *z*+1/2; (ii) *x*, *y*−1, *z*.
